# Novel cryo-EM structure of an ADP-bound GroEL–GroES complex

**DOI:** 10.1038/s41598-021-97657-x

**Published:** 2021-09-14

**Authors:** Sofia S. Kudryavtseva, Evgeny B. Pichkur, Igor A. Yaroshevich, Aleksandra A. Mamchur, Irina S. Panina, Andrei V. Moiseenko, Olga S. Sokolova, Vladimir I. Muronetz, Tatiana B. Stanishneva-Konovalova

**Affiliations:** 1 Faculty of Biology, Lomonosov Moscow State University, Moscow, Russia; 2 Faculty of Bioengineering and Bioinformatics, Lomonosov Moscow State University, Moscow, Russia; 3 National Research Center «Kurchatov Institute», Moscow, Russia; 4 Petersburg Nuclear Physics Institute Named by B.P. Konstantinov of NRC «Kurchatov Institute», 1, Orlova Roshcha, Gatchina, Russia 188300; 5 Shemyakin-Ovchinnikov Institute of Bioorganic Chemistry, Russian Academy of Sciences, Moscow, Russia; 6 Belozersky Institute of Physico-Chemical Biology, Lomonosov Moscow State University, Moscow, Russia

**Keywords:** Chaperones, Cryoelectron microscopy

## Abstract

The GroEL–GroES chaperonin complex is a bacterial protein folding system, functioning in an ATP-dependent manner. Upon ATP binding and hydrolysis, it undergoes multiple stages linked to substrate protein binding, folding and release. Structural methods helped to reveal several conformational states and provide more information about the chaperonin functional cycle. Here, using cryo-EM we resolved two nucleotide-bound structures of the bullet-shaped GroEL–GroES_1_ complex at 3.4 Å resolution. The main difference between them is the relative orientation of their apical domains. Both structures contain nucleotides in cis and trans GroEL rings; in contrast to previously reported bullet-shaped complexes where nucleotides were only present in the cis ring. Our results suggest that the bound nucleotides correspond to ADP, and that such a state appears at low ATP:ADP ratios.

## Introduction

A newly synthesized polypeptide chain should fold in a specific way to form a native structure. Only then does the protein acquire its functional properties. Correct protein folding both in vivo and in vitro could be disrupted by intermolecular interactions, which could cause the formation of aggregates, since the intracellular concentration of macromolecules may be more than 300 mg/mL^1^. Under such conditions, multidomain proteins could fold properly only in the presence of specific cellular assistants that are not part of the native protein: “molecular chaperones”^2^. Subsequently, the term “molecular chaperones”, or simply “chaperones”, began being applied to large families of proteins that are involved in the folding, ensemble formation, and translocation of macromolecules, but are not involved in the realization of their function. They are divided into six large groups, depending on their size. Five of them—Hsp 100, Hsp 90, Hsp 70, Hsp 40 and chaperonins (Hsp 60), which form large multisubunit complexes of 800–900 kDa—have slow ATPase activity. And the sixth group—small heat shock proteins (12–43 kDa)—work in an ATP-independent manner^3,4^.

Members of the chaperone family are present in the cells of bacteria^5^, archaea^6^, eukaryotes^7^ and are even encoded in viral genomes^8–10^. The most studied chaperonin GroEL–GroES complex is responsible for protein folding in bacterial cells. This chaperonin is involved not only in assisting the folding of newly synthesized polypeptides, but also in preventing aggregation of proteins under heat shock and repairing proteins that were damaged or misfolded by other stress conditions.

In the first studies of the functional activity of the GroEL–GroES complex, it was shown that about 10% of all cytoplasmic proteins from *E. coli* called for its assistance for proper work under normal growth conditions^11,12^. However, further research claimed that only 5% of *E. coli* proteins require obligate GroEL–GroES help to form a native conformation^13,14^, and about 10% proteins require both the GroE and DnaK systems simultaneously, where GroEL acts downstream^15,16^. GroEL is composed of 14 identical subunits with a molecular weight of 60 kDa combined into two seven-membered rings. Each of the GroEL subunits consists of three domains: apical, which is designed to bind non-native proteins and co-chaperonin GroES, intermediate and equatorial with an ATP-binding pocket. The rings are stacked from the equatorial domains’ side forming a barrel-like structure. The apical and intermediate domains of GroEL subunits form the walls of the large central cavity, while the equatorial domains form its deck. There is one cavity per each ring. The inner surface of these cavities is lined with non-polar amino acids^17–19^. In addition, 7 subunits of co-chaperonin GroES with a molecular weight of 10 kDa each form a dome-shaped complex that is able to bind to the apical domains of GroEL, closing the hydrophobic cavity. This provides rearrangements, which increase the hydrophilicity of the chamber to establish conditions suitable for substrate protein folding^20,21^. The chaperonin complex recognizes its molecular substrates if their peptide sequence contains one or more mobile loop–like hydrophobic patches^22^. However, there is no evidence of whether the number of hydrophobic sites affects the chaperonins’ efficiency of substrate binding. It has also been shown that a significant part of GroEL substrates contain—TIM-barrel domains^14,23^.

Previously, it was considered that the chaperonin cavity played a passive role in protein folding by just isolating it from the cellular environment^24^. However, now it is thought to play an active role: chaperonin functioning requires conformational rearrangements which happen in an ATP-dependent manner and allow for efficient substrate protein binding, folding and release^25^. Moreover, recent studies show the importance of the flexible C-terminal regions of GroEL subunits for substrate binding, encapsulation and retention within the cage^26–28^.

Structural studies provided valuable information about GroEL–GroES functional cycle. As revealed by a 2.8 Å-resolution X-ray structure, inter-subunit contacts in each ring are provided by hydrophobic interactions in the equatorial domains and salt bridges in the intermediate and apical domains^17^. Contacts between rings are formed by both electrostatic and hydrophobic interactions, especially by salt bridges E461–R452 and hydrogen bond K105-A109^29–31^. Importantly, the occupancy of the ATP-binding pockets affects the inter-ring interactions. As revealed by the X-ray structure of an ATP-bound GroEL, ATP-binding to one ring causes rearrangements in its salt bridges and causes concerted rotations of its apical domains^18,32^. This rotation leads to a conformation that is capable of GroES binding. In the normal course of functioning, GroES binding happens after substrate binding and results in the encapsulation of the substrate protein. Whether this set of events occurs in both rings simultaneously or one at a time has been a topic of long-time debate^33^. A “football cycle” model suggests that the rings work simultaneously (i.e. GroES heptamers bind to both ends of the barrel: leading to the formation of a football-shaped complex)^34^, while the “bullet cycle” suggests that the rings work in turns via negative inter-ring cooperation^35^. Both types of structures have been crystallized, which supports each model^20,36^. Some studies suggest that a bullet-shaped state is more physiologically relevant, as it appears at a physiological concentration ratio of ATP:ADP^37,38^. Others assume that there are two possible cycles: asymmetric and symmetric^34^. The asymmetric cycle occurs in the absence of the substrate protein. It begins with a football-shaped GroEL–GroES_2_ complex carrying 14 ATP, which hydrolyse over time. However, hydrolysis in one ring occurs slightly faster than in another, which leads to the formation of a bullet-shaped GroEL–GroES complex and chaperonin continues to function in this state. Vice versa, the symmetric cycle is observed in the presence of substrate and the higher its concentration is—the more football-shaped complexes will be formed^39,40^. In addition, a recent study suggested that ring separation may be a part of the GroEL–GroES complex working cycle^41^. It occurs after ATP hydrolysis in the *cis*-ring and ATP binding to the *trans* ring and could be crucial for the substrate release.

A revolution in cryo-EM^42^ allowed this method to shed new light on the functional studies of GroEL–GroES machinery. For instance, it allowed to characterize conformational variations within subunits in GroEL^43^ and advance studies of GroEL–GroES interactions with substrate proteins^27,44^. However, all of the existing cryo-EM structures of GroEL–GroES have moderate resolutions ranging from 7.7 to 15.9 Å^27,44^. In this work, with the use of cryo-EM, we resolved two conformations of the bullet-shaped GroEL–GroES complex at 3.4 Å. These conformations exhibit differences in the relative orientations of the apical domains of the trans ring. The local resolution of the equatorial domains allowed us to observe extra densities in the ATP-binding pockets of both rings. The presence of nucleotides in both rings of the bullet-shaped complex distinguishes our structures from previously reported GroEL–GroES complexes, suggesting that they represent a new stage of the functional cycle.

## Results and discussion

### The transition from football- to bullet-shaped complexes observed by EM

**Figure 1:**
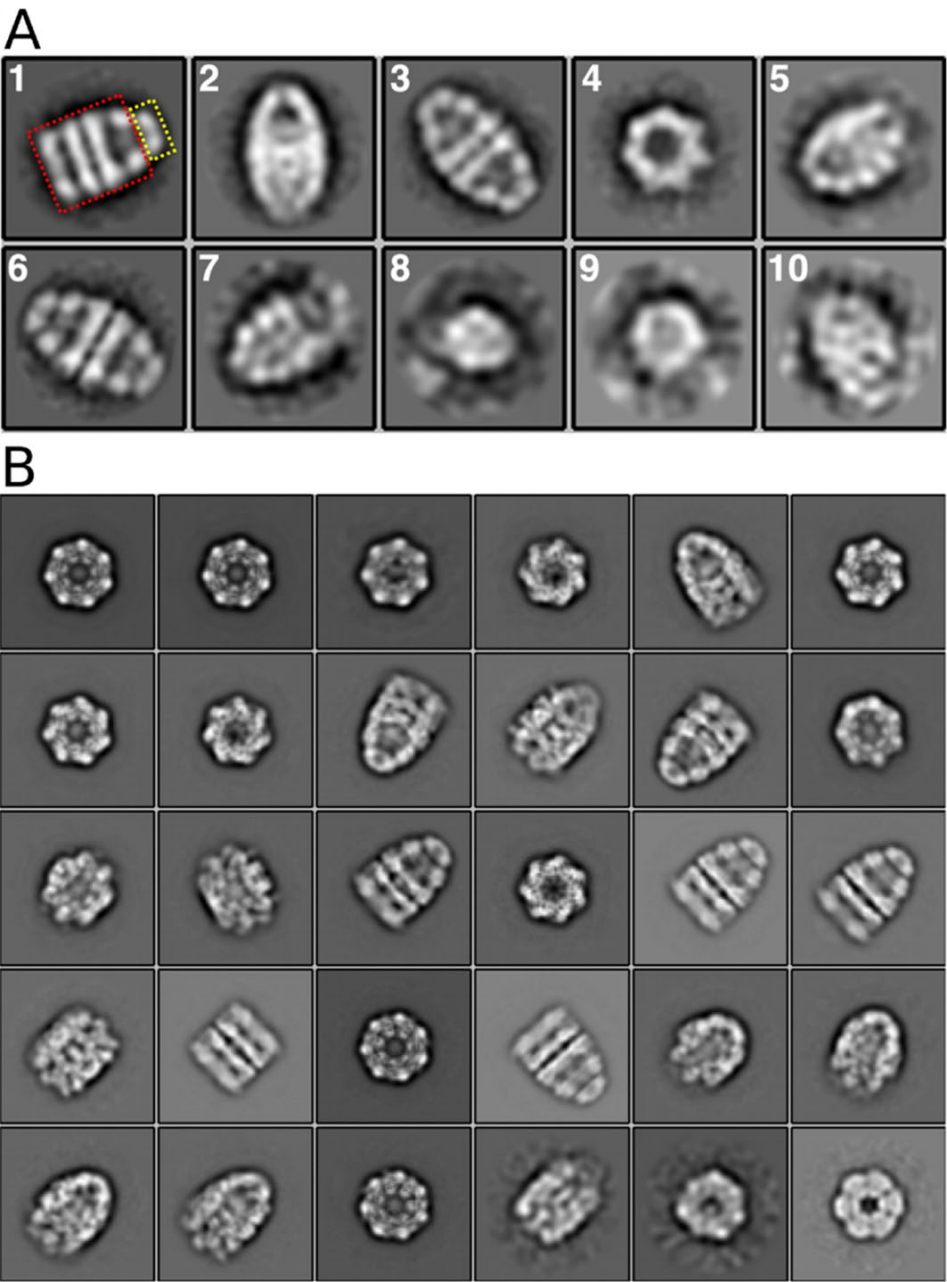
2D class averages from the negatively stained (**A**) and from the cryo-EM (**B**) GroEL–GroES sample.

A GroEL–GroES sample was first examined by negative stain EM. 2D classification of selected particles indicated the presence of both bullet- and football-shaped complexes (Fig. 1A). However, football-shaped complexes were absent on 2D class averages from cryo-EM samples, which were frozen after 10× concentration and additional incubation for 30 min (50 min total incubation) (Fig. 1B) (see “Methods” section for data collection and image processing procedures).

Our results indicate that after 20 min of incubation, GroEL–GroES passes through the symmetric cycle. However, after concentration and additional incubation for 30 min only the asymmetric cycle remains. The simplest explanation for this observation is the change of the cycling regime caused by the depletion of the ATP in the sample. Football-shaped particles were observed in the sample with the ADP:ATP ratio close to 1:8, but after a total incubation procedure the ratio shifts to a 5:1 value and, in such conditions, only the asymmetric cycle takes place (see Supplementary information section “ATP/ADP concentration in the samples”). An alternative explanation of the observation is related to the action of the hypothetical substrate: studies carried out in the GH Lorimer laboratory in 2013 showed that symmetric complexes persisted for tens of minutes in the presence of a substrate protein in the reaction mixture, while in its absence football-shaped particles eventually turned into bullet-shaped^34,40^. Data obtained by SDS PAGE indicated the purity of our protein samples (Supplementary Fig. S1), however, we assumed that our GroEL samples could contain a certain amount of denatured monomers, which could not be distinguished from native subunits using SDS PAGE. As it has been previously suggested, GroEL is capable of participating in self-folding^45^. Thus, monomers could serve as substrates for assembled GroEL–GroES complexes, which would explain the appearance of football-shaped particles during the short incubation (20 min) of GroEL with GroES, prior to negative staining (see “Methods” section for details). Further incubation and self-folding led to the disappearance of the free substrate; therefore, football-shaped complexes were absent in the sample by the time of vitrification.

### Two conformations of the GroEL–GroES_1_ complex

**Figure 2:**
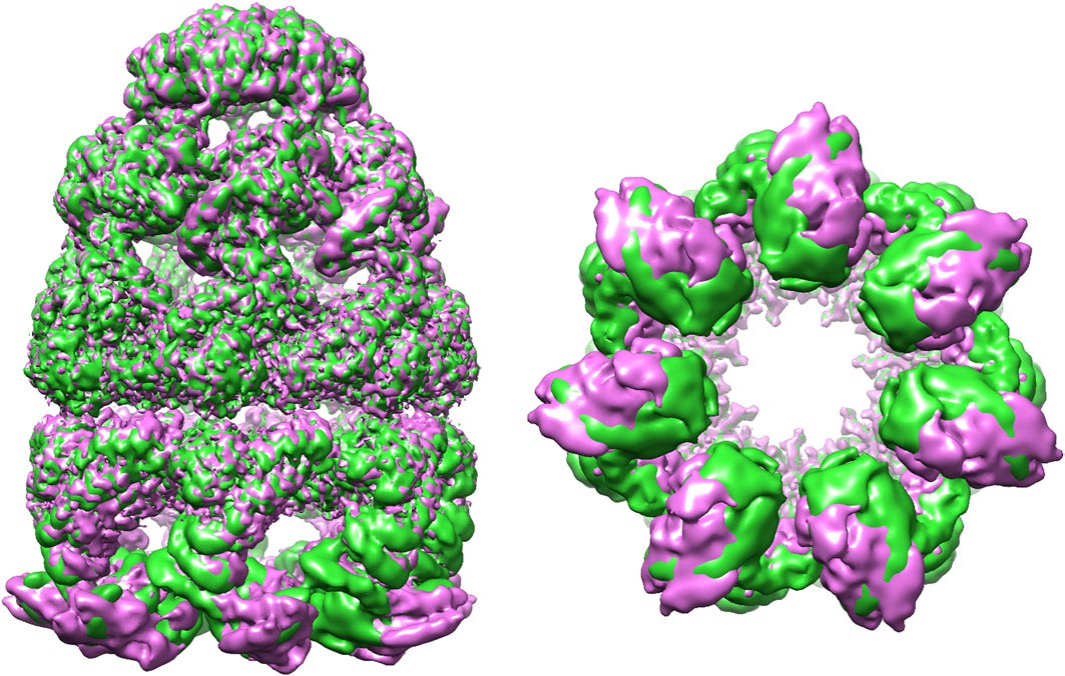
Cryo-EM density maps of the “tight” conformation (green) and the “wide” conformation (pink).

The results of the 2D classification of cryo-EM images demonstrate the presence of bullet-shaped GroEL–GroES complexes, as well as free GroEL and GroES particles (Fig. 1B). To explore the conformational landscape of the GroEL–GroES complexes, 3D classification was performed with and without C7 symmetry imposed. After additional refinement, we have solved two major classes with different orientations of the apical domains in the trans ring. In the first structure, the apical domains are located further away from the symmetry axis than in the second one, and, therefore, we denoted them as “wide” and “tight” (Fig. 2). The estimated resolution was 4.0 Å (C1)/3.4 Å (C7) and 4.24 Å (C1)/3.4 Å (C7) for the “wide” and “tight” states, respectively (Supplementary Fig. S2). While a certain degree of symmetry-mismatch in the apical domains of the trans ring was observed in the symmetry-free (C1) structures, extensive comparison of the maps showed that this difference cannot be reliably interpreted at the given resolution. Furthermore, as we were interested in the exact mode of the nucleotide binding to both rings, our analysis benefited from the higher resolution C7 maps. To address the uncertainty regarding the usage of C7 symmetry, we decided to investigate further whether we are dealing with two discrete conformations or a mix of conformations. We have performed 3D Variability analysis in cryoSPARC with 3 eigenvectors and a 6 Å low pass filtering. The resulting distribution of the particles over principal components does not show a discrete distribution. However, the reconstructions from the first eigenvector, representing the largest motion, essentially represent a morphing between the “wide” and “tight” classes (Supplementary Movie 1).

### Bullet-shaped complexes contain nucleotides in both rings

**Figure 3:**
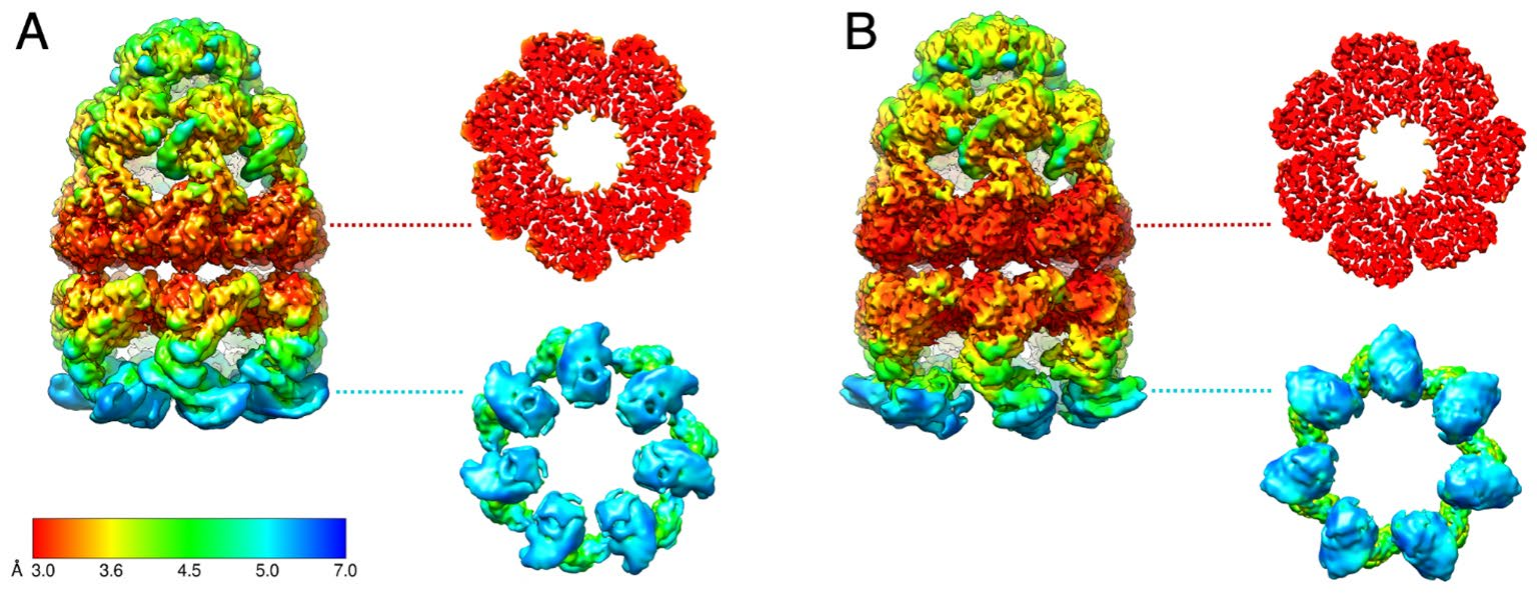
Cryo-EM structures (C7) of the bullet-shaped GroEL–GroES complex coloured according to local resolution. “Tight” (**A**) and “wide” (**B**) conformations are shown with additional slices through equatorial and apical domains.

**Figure 4:**
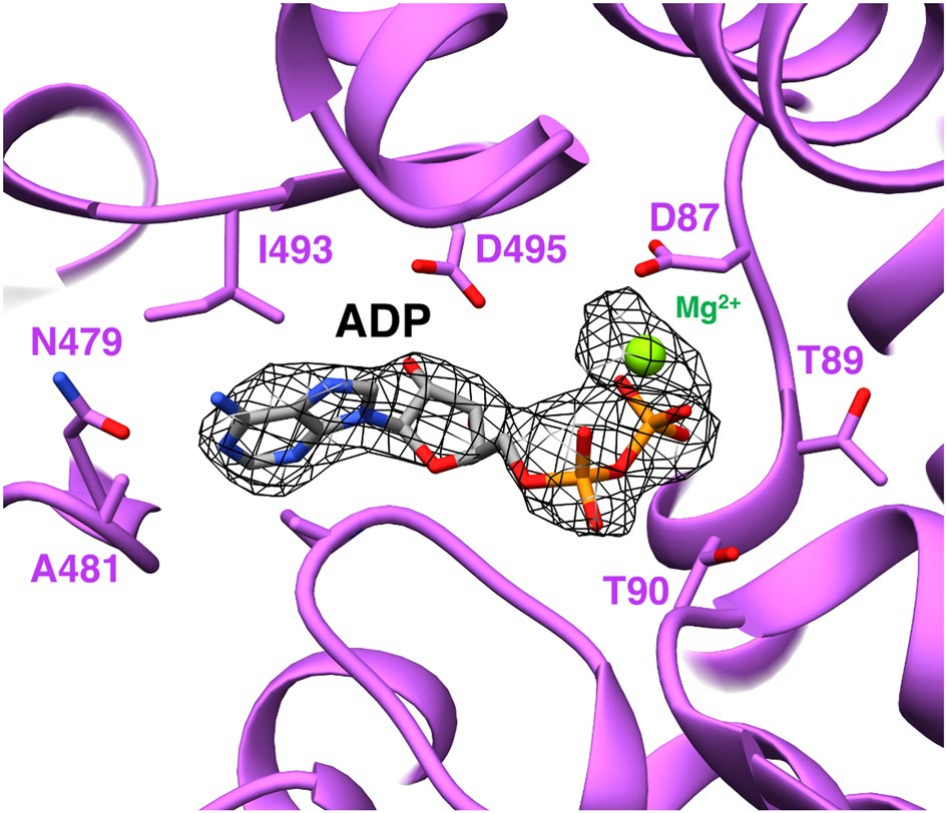
Density in the nucleotide-binding pocket (mesh) in a wide-conformation trans-ring with an atomic model of bonded ADP and Mg2+.

Figure 3 shows the surface and the slices through the C7 structures coloured according to local resolution. The highest value (about 3.0 Å) is observed in the regions of the equatorial domains that are responsible for inter-ring contacts and ATP-binding. At this resolution, we can conclude with confidence that the subunits of both rings contain nucleotides in the nucleotide-binding pockets, most likely corresponding to ADP (Fig. 4, Supplementary Figs. S4, S5). To our knowledge, these are the first reported bullet-shaped structures with nucleotide presence in both rings.

All GroEL–GroES_2_ (football) structures reported to date were obtained using X-ray crystallography at resolutions 3.6–3.8 Å (Table 1). The nucleotide-binding pockets of both rings were occupied either by ATP or by its analogue ADP-BeF_x_. GroEL–GroES_1_ (bullet) complexes were resolved by both X-ray crystallography (with resolutions from 2.8 to 3 Å) and cryo-EM (7.7–15.9 Å). For X-ray structures of bullet forms, the presence of nucleotides (ADP or ADP-Mg-AlF_3_) was detected in the GroES-capped (cis) ring. In some cryo-EM bullet complexes, the ADP was also placed in the cis ring, while in other structures nucleotides were not modelled, due to resolution limitations.

**Table 1:** Structures of the GroEL–GroES complex deposited in the Protein Data Bank.

PDB id	Method	Form	Ligands in the cis ring	Ligands in the trans ring	Resolution (Å)
5OPX	X-ray	Football	ADP–Mg–BeF–K	ADP–Mg–BeF–K	3.64
3WVL	X-ray	Football	ATP–Mg–K	ATP–Mg–K	3.788
4PKN	X-ray	Football	ADP–Mg–BeF–K	ADP–Mg–BeF–K	3.66
4PKO	X-ray	Football	ADP–Mg–BeF–K	ADP–Mg–BeF–K	3.84
4V4O	X-ray	Bullet	ADP–Mg	–	2.8
1SVT	X-ray	Bullet	ADP–Mg–AlF3–K	–	2.808
1SX4	X-ray	Bullet	ADP–Mg	–	3
1PF9	X-ray	Bullet	ADP–Mg	–	2.993
1PCQ	X-ray	Bullet	ADP–Mg–AlF3–K	–	2.808
1AON	X-ray	Bullet	ADP–Mg	–	3
3ZPZ	EM	Bullet	ADP–Mg	–	8.9
3ZQ0	EM	Bullet	ADP–Mg	–	9.2
3ZQ1	EM	Bullet	ADP–Mg	–	15.9
2C7C	EM	Bullet	–	–	7.7
2C7D	EM	Bullet	–	–	8.7
1GRU	EM	Bullet	–	–	12.5

**Figure 5:**
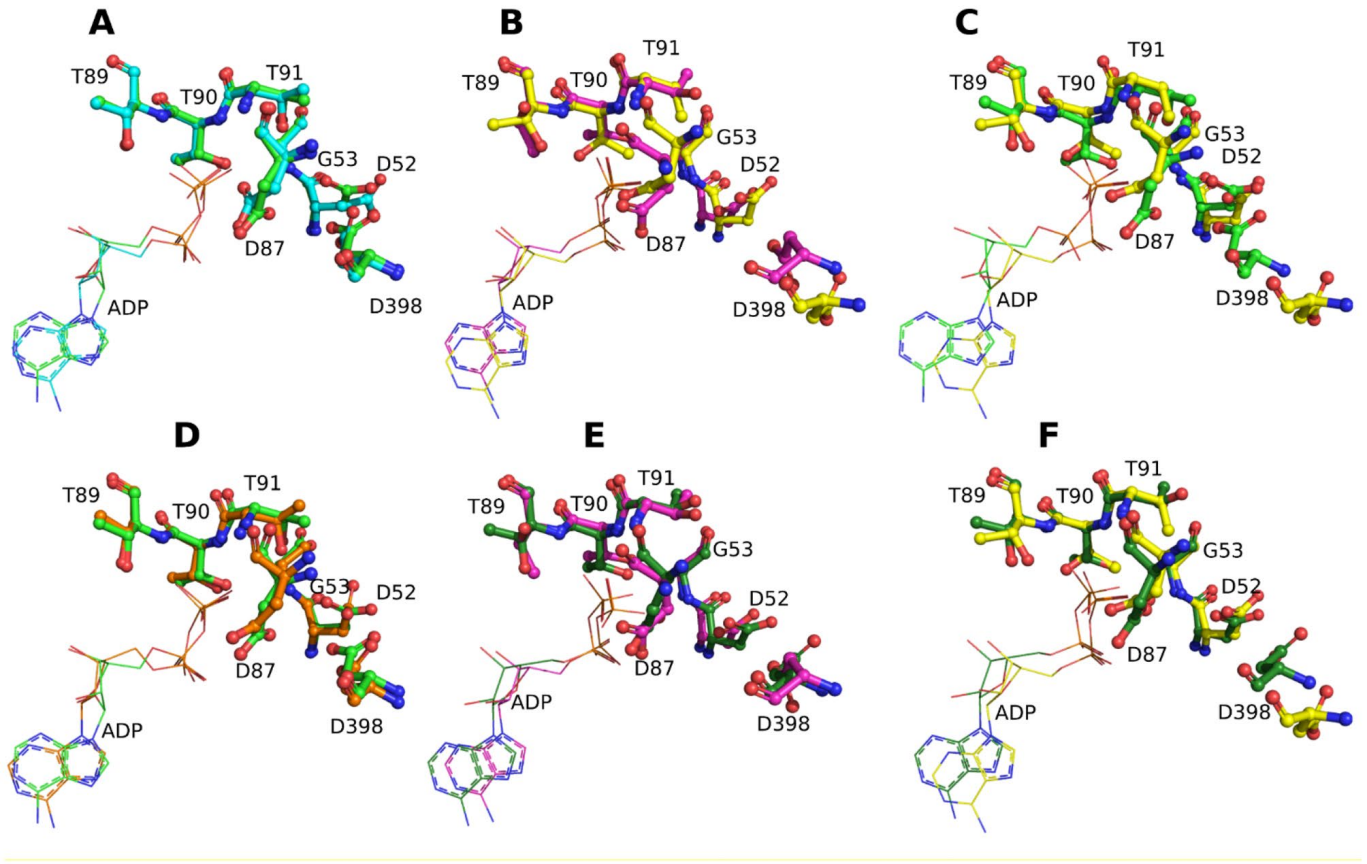
Comparison of the ATP-binding pockets: (**A**) tight cis ring (light green) vs wide cis ring (blue); (**B**) tight trans ring (yellow) vs wide trans ring (magenta); (**C**) tight cis ring (light green) vs tight trans ring (yellow); (**D**) tight cis ring (light green) vs R′′ ADP-bound-site 4V4O (orange); (**E**) wide trans ring (magenta) vs R-ADP ADP-bound-site 4KI8 (dark green); (**F**) tight trans ring (yellow) vs R-ADP ADP-bound-site 4KI8 (dark green). For additional comparison of tight, wide and R-ADP conformations refer to the Supplementary materials section “Wide and tight conformations of GroEL–ADP14–GroES1 comparison”.

The current classification of the GroEL–GroES complex contains several states for the single ring: T—apo-form, R—ATP-bound form, R′—ATP-bound ring associated with GroES, R′′—ADP-bound ring associated with GroES, R-ADP—ADP-bound ring after GroES dissociation^46,47^. According to this classification, here we observe the R′′/R-ADP state for both tight and wide conformations. Next, we studied the relative orientation of amino acid residues in the nucleotide-binding pockets in more detail. When they are occupied by ATP, residues D52, G53, T89, T90, and D398 are bound to the APT gamma-P group, and the D87 is bound to the Mg(2+)^48^. We compared the location of these residues in our tight and wide conformations with their location in the ADP-bound R′′ and R-ADP references. The cis ADP-bound ring of the PDB id: 4v4o was used as a R′′ reference, and the structure of the PDB id: 4ki8 as a R-ADP reference. The assessment shows almost no difference between nucleotide-binding pockets of the cis rings of the tight and wide conformations (Fig. 5A). The configuration of the nucleotide-binding pocket in trans-rigs is slightly different for the D398 (Fig. 5B). Diversity in the nucleotide-binding pockets is much more pronounced in cis- and trans-rings (Fig. 5C). The configuration for the cis-rings is similar to R′′-conformation (ADP-bound cis-ring in the GroEL–GroES_1_ complex) (Fig. 5D). The configuration of the ATP-binding pocket of the trans-ring in wide conformation is very similar to the R-ADP state (Fig. 5E) and it differs in the orientation of D398 for tight conformation, nevertheless preserving much similarity (Fig. 5F).

**Figure 6:**
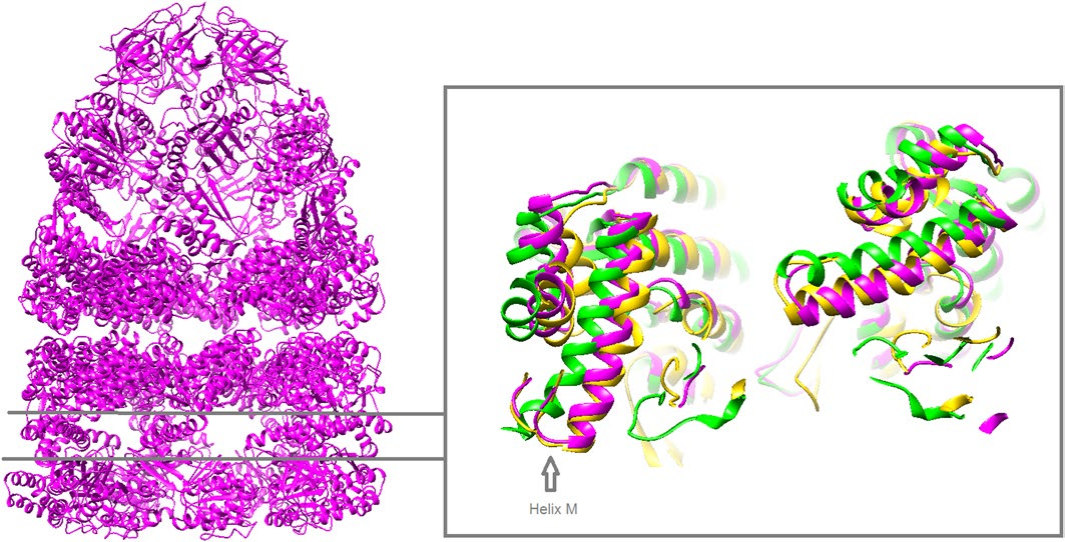
Comparison of the wide and tight conformations. Atomic model of the wide conformation (left) and a slice through the intermediate domains (right). On the right, the wide conformation (magenta) is aligned with the tight conformation (green) and 2c7d (gold); helix M of the intermediate domain is labelled.

Next, we compared our atomic models with the atomic models deposited in the Protein Data Bank (Supplementary Table S2) to elucidate to which stage of the ATPase cycle the tight and wide conformations could belong to. The conformations of the trans ring subunits were of particular interest: while the tight conformation matched several models, the wide conformation only aligned well with the structure of the GroEL-ADP_7_-GroES1 complex (PDB ID: 2c7d), presented in Ranson et al.^49^ (Fig. 6). This structure corresponds to the step of the functional cycle after the ATP hydrolysis in the cis ring and prior to the ATP binding to the trans ring. Despite the similarity of these structures, 2c7d lacks nucleotides in the trans ring. However, the resolution of the corresponding cryo-EM structure was 8.70 Å, which would not allow to clearly state the presence or the absence of nucleotides. The same study also presented the cryo-EM structure of the ATP_7_–GroEL–ADP_7_–GroES_1_ complex at a lower resolution—a step after ATP binding to the trans ring (EMD-1046). At this level, it is not possible to find out whether one of our two structures coincided with the structure of EMD-1046. If we do not take into account the difference in the nucleotide states of the trans rings, then the wide isoform would correspond to a time-extended stage when the GroEL–ADP_7_–GroES complex begins to bind ATP molecules in the trans ring, but the apical domains of the trans ring have not rotated yet (i.e. between the steps depicted in Fig. 6d,e of Ranson et al.)^49^. Then, the tight isoform might be the next step of the cycle (Fig. 6e of Ranson et al.).

The results obtained from our negatively stained samples indicated the occurrence of a symmetric cycle of the GroEL–GroES association at the beginning. The cryo-EM samples were prepared after enough time for most of the ATP to be hydrolysed to the ADP; by this moment, no football-shaped complexes were left. Thus, under such conditions and in the absence of a substrate protein, the system only works in an asymmetric cycle.

The obtained results indicate that with a high ADP:ATP [5:1] ratio (Supplementary Fig. S8) and without an unfolded substrate, only an asymmetric GroEL–GroES cycle takes place. The fact that the overwhelming majority of the GroEL–GroES_1_ particles presented in the sample belong to the R′′/R-ADP class suggests that at such conditions the state with both rings occupied by ADP is a limiting stage of the asymmetric cycle. Additionally, the same observation diminishes the significance of the T (apo-GroEL) state in the cycle. This is also supported by the nucleotide content of the GroEL particles free from GroES in our sample (see Supporting Information section “GroEL structure obtained with Cryo-EM”). According to our evaluation, these free GroEL particles represent the second limiting stage of the same asymmetric cycle belonging to the R/R-ADP state. We believe that the only probable route to the R′′/R-ADP state passes through the nucleotide-bound states: R′′/R-ADP evolves to R-ADP/R-ADP after GroES dissociation, then the direct substitution of ADP to ATP leads to the R/R-ADP state and the association with new GroES leads to the subsequent R′/R-ADP and, finally, the R′′/R-ADP. The lack of the T state GroEL in the studied samples supports this hypothesis. In other words, the R-ADP state evolves to form the R state, skipping the T state, by the direct substitution of ADP to ATP.

## Conclusion

The GroEL–GroES complex undergoes many conformational changes during its functional cycle. Cryo-EM allows for the identification of conformations at different steps of the cycle, and here we resolved two bullet-shaped complexes at 3.4 Å resolution. Both structures contain ADP nucleotides in cis and trans rings, but conformations of the trans rings differ. We propose that such complexes appear under low ATP:ADP ratios, which precludes the substitution of the ADP for the ATP in the trans ring and the subsequent release of GroES from the cis ring. The differences between the trans rings of the two resolved structures may result in distinct behaviour in the matter of nucleotide binding and release, which should be revealed by future studies.

## Methods

### Purification of chaperonin GroEL and co-chaperonin GroES

*E. coli* cells (strain W3110) were transformed with the pOF39 plasmid that encodes GroEL and GroES. The cells were grown in LB medium in the presence of ampicillin (50 μg/mL). Extraction, sulfate ammonium fractionation, and DEAE-Sephacel ion-exchange chromatography were performed as described by Corrales and Fersht^50^. The proteins were eluted with a 0–500 mM NaCl gradient in a buffer containing 50 mM Tris, 2 mM DTT, and 0.1 mM EDTA, pH 7.2. GroES was eluted at 0.13–0.25 M NaCl and GroEL was eluted at 0.33–0.38 M NaCl. The fractions containing GroEL were rapidly heated to 58 °C and then cooled to 25 °C; further, Mg^2+^-ATP (pH 7.0) was added to the final concentration of 2 mM and the solution was again incubated for 20 min at 58 °C. After that, GroEL was re-chromatographed on the DEAE-Sephacel, under the same conditions. Pure fractions were concentrated using Centriprep centrifugal filters, and then dialyzed against 10 mM of Tris–HCl buffer, pH 7.5. The fractions containing GroES were pooled and heated at 60 °C for 20 min, and then the precipitate was removed. The procedure was repeated with heating to 80 °C. The resulting solution of GroES was concentrated using Centriprep centrifugal filters and then dialyzed against 10 mM of Tris–HCl buffer, pH 7.5. The obtained preparations of GroEL and GroES were stored in 80% ammonium sulfate at + 4 °C.

Concentrations of GroEL_14_ and GroES_7_ were determined spectrophotometrically considering that the molar extinction coefficients were 1.68 × 10^5^ M^−1^ cm^−1^ and 1.04 × 10^4^ M^−1^ cm^−1^, respectively^51^.

For negative staining TEM, the GroEL–GroES complex was prepared by incubating 1 μM GroEL with 3 μM GroES in 50 mM of Tris–HCl buffer (pH 7.5) containing 10 mM KCl, 10 mM MgCl_2_ and 3 mM ATP for 20 min at 20 °C. Cryo-EM samples were prepared from the previously mentioned sample by concentrating it 10 times with a 100 kDa concentrator and additionally incubating for 30 more min at 20 °C.

### Cryo-EM data collection

For grid preparation, 3 μL of the sample was applied to glow-discharged electron microscopy grids (Quatifoil R1.2/1.3) and plunge-frozen in liquid ethane using the FEI Vitrobot Mark IV at 4.5 °C. 6784 movies were collected using the Titan Krios electron microscope equipped with the Falcon II electron detector with the pixel size of 1.107 Å. 25 frames were recorded per exposure with the dose of 4e/Å^2^ per movie frame. Motion correction, CTF estimation, and particle picking were performed in Warp^52^. Particles were exported to Relion^53^ for 2D classification. Initial model generation, consecutive 3D classification/refinement with no symmetry imposed (C1) and with C7 symmetry were performed in cisTEM^54^ resulting in two classes representing different states, each containing approximately 42.000 particles. Particles from both classes were imported to cryoSPARC^55^, refined against structures from cisTEM without symmetry and with a C7 symmetry applied. Resolution of 3.4 Å (C7) and 4.0 Å (C1) for the “wide” structure and 3.4 Å (C7) 4.2 Å (C1) for the “tight” structure was estimated. Finally, both C1 and C7 structures were analysed for local resolution variations and locally sharpened in cryoSPARC. 3D Variability analysis^56^ was performed to analyse the mode of apical domain’s motion with the following parameters: low pass filtering = 6 Å, 3 modes.

### Model building

The crystal structure of GroEL–GroES-ADP7^45^ (PDB: 1SX4) was used as an initial reference. Atomic models were built and refined using ISOLDE^57^, Coot^58^, and Phenix^59^. The full-size model was created with Chimera^60^ using C7 symmetrized maps. Energy minimization was also carried out using GROMACS 2020^61^. Fitting of ligands was carried out on the basis of the article^62^.

## Supplementary Information

Supplementary Information.

Supplementary Movie 1.

## Data Availability

The 3D cryo-EM density maps have been deposited in the Electron Microscopy Data Bank (accession no. EMD-13293 for the “wide” conformation and no. EMD-13308 for the “tight” conformation). The atomic coordinates have been deposited in the Protein Data Bank (PDB ID codes 7PBJ and 7PBX for the “wide” and the “tight” conformations, respectively).
