# Supplementary material for: Novel cryo-EM structure of an ADP-bound GroEL–GroES complex
Source: Sci Rep. 2021 Sep 14;11:18241. doi: 10.1038/s41598-021-97657-x (PMC8440773; doi:10.1038/s41598-021-97657-x)
Supplement: Supplementary file 1 — Supplementary Information. [file 41598_2021_97657_MOESM1_ESM.doc]

**Novel cryo-EM structure of an ADP-bound GroEL-GroES complex**

Sofia S. Kudryavtseva^1,2^, Evgeny B. Pichkur^3,4^, Igor A. Yaroshevich^1^, Aleksandra A. Mamchur^1^, Irina S. Panina^5^, Andrei V. Moiseenko^1^, Olga S. Sokolova^1^, Vladimir I. Muronetz^2,6^, Tatiana B. Stanishneva-Konovalova^1*^

^1^ Faculty of Biology, Lomonosov Moscow State University, Moscow, Russia

^2^ Faculty of Bioengineering and Bioinformatics, Lomonosov Moscow State University, Moscow, Russia

^3^ National Research Center «Kurchatov Institute», Moscow, Russia

^4^ Petersburg Nuclear Physics Institute Named by B.P. Konstantinov of NRC «Kurchatov Institute», 1, Orlova Roshcha, 188300 Gatchina, Russia

^5^ Shemyakin-Ovchinnikov Institute of Bioorganic Chemistry, Russian Academy of Sciences, Moscow, Russia

^6^ Belozersky Institute of Physico-Chemical Biology, Lomonosov Moscow State University, Moscow, Russia

*corresponding author

email: [stanishneva-konovalova@mail.bio.msu.ru](mailto:stanishneva-konovalova@mail.bio.msu.ru)

**Supplementary information**


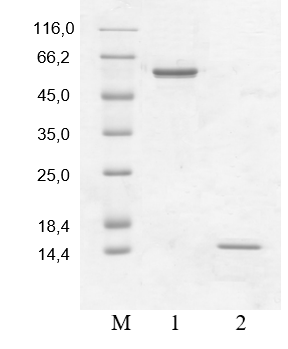


*Supplementary Figure S1. SDS PAGE of protein molecular weight markers (M), purified GroEL (1) and GroES (2) samples.*


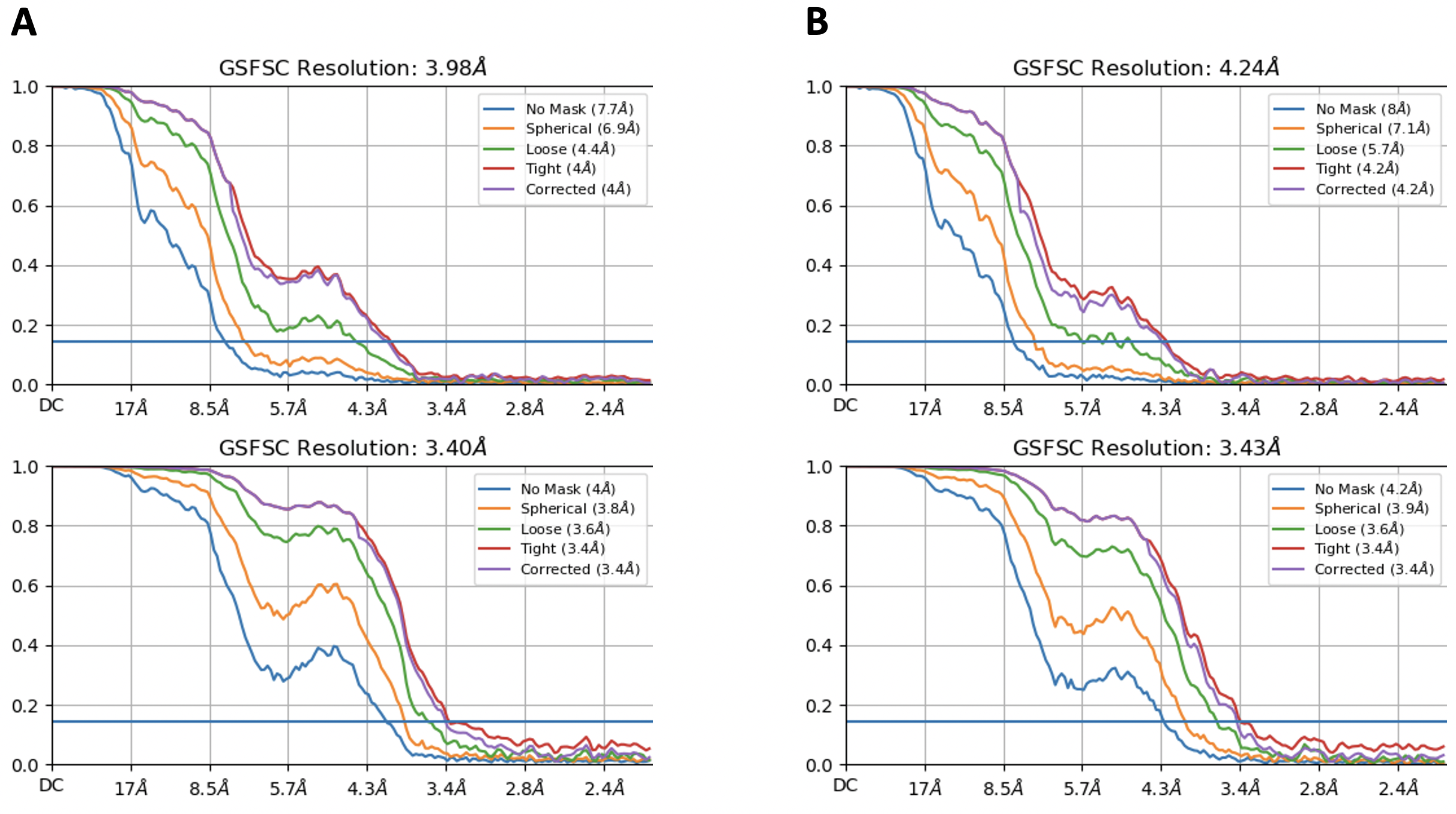


*Supplementary Figure S2. Average resolution estimated for C1 (top) and C7 (bottom) maps of the “tight” (A) and “wide” (B) conformations of the GroEL-GroES complex.*

*
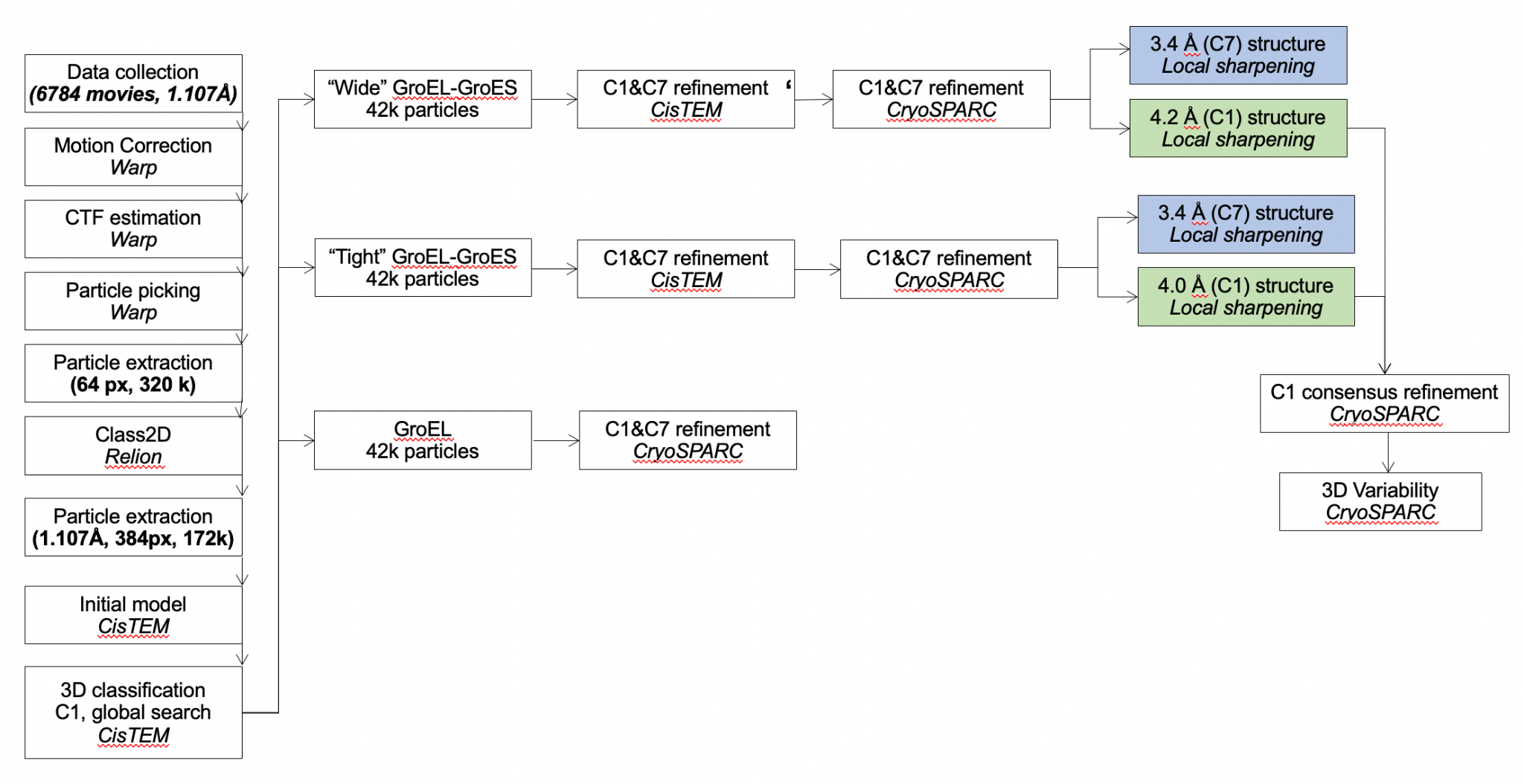
*

*Supplementary Figure S3. Cryo-EM data collection and processing.*

**Fitting ATP vs ADP in GroEL-AXP14-GroES1 complexes**

*
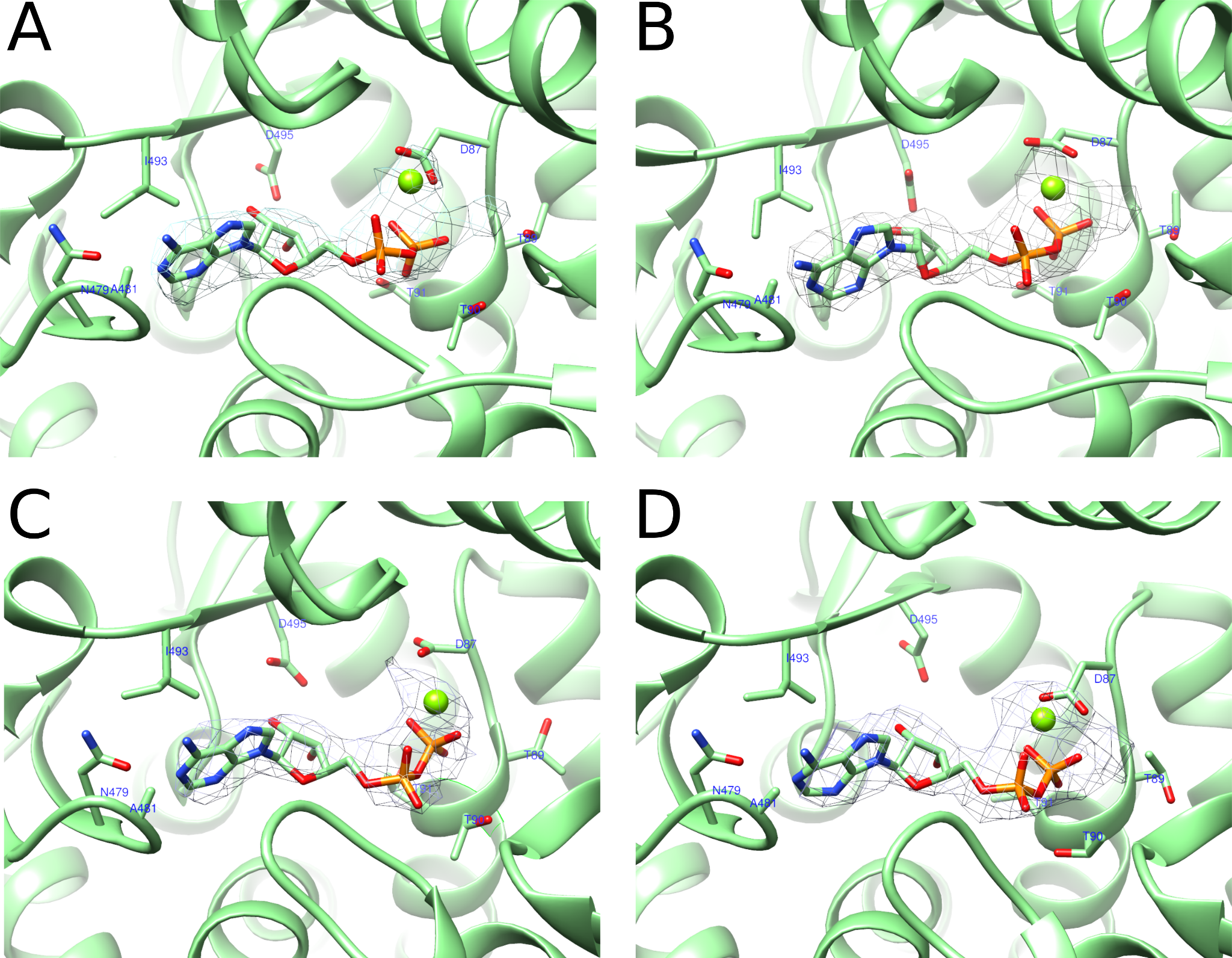
*

*Supplementary Figure S4. Fitting ADP and Mg^2+^ into the density located in the nucleotide binding pocket (mesh) for A - wide conformation cis-ring; B - tight conformation cis-ring; C - wide conformation trans-ring; B - tight conformation trans-ring.*

*
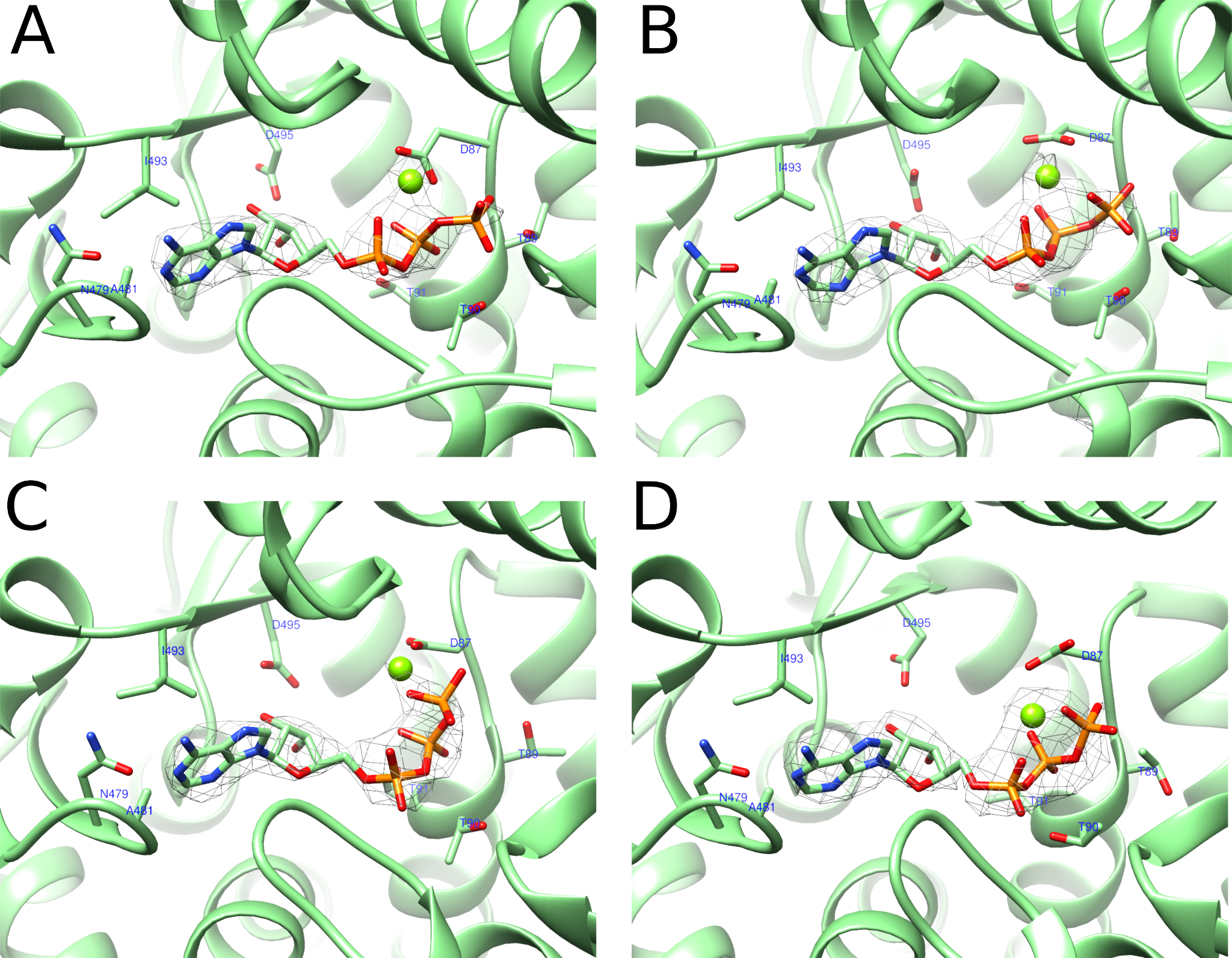
*

*Supplementary Figure S5. Fitting ATP and Mg^2+^ into the density located in the nucleotide binding pocket (mesh) for A - wide conformation cis-ring; B - tight conformation cis-ring; C - wide conformation trans-ring; B - tight conformation trans-ring.*

According to the obtained density maps for GroEl-GroES_1_ complexes, it is obvious that all nucleotide-binding pockets contain ligands, so the structures should, without a doubt, be classified as GroEL-AXP_14_-GroES_1_. To address the question if bonded nucleotides are ATP or ADP molecules, the local cross-correlation coefficients for both ATP and ADP models were calculated. To determine the correlation coefficient, regions of structure and density were cut off with 1 nm distance from the nucleotide. Then, the local correlation between them was calculated using the PHENIX software package. Calculated correlation coefficients are: 0,75 - for cis-ring ADP-wide (S4 A), 0,67 - for cis-ring ADP-tight (S4 B), 0,76 - for trans-ring ADP-wide (S4 C), 0.65 - for trans-ring ADP-tight (S4 D), 0,73 - for cis-ring ATP-wide (S5 A), 0,63 - for cis-ring ATP-tight (S5 B), 0,72 - for trans-ring ATP-wide (S5 C), 0,62 - for trans-ring ATP-tight (S5 D). This analysis points in favour of assigning both tight and wide conformations to the GroEL-ADP14-GroES1 class.

**Wide and Tight conformations of GroEL-ADP_14_-GroES_1_ comparison**

The trans-rings of the tight and wide conformations differ from each other predominantly through the intermediate and apical domains positions relative to the equatorial domain. First, we aligned the trans-rings (RMSD = 2.97 Å) and calculated the main component of the domain's principal axes (S6, right). The domain's arrangement can be characterized by the angle defined between the centres of mass of the backbone atoms of each domain (S6, left), which are equal to 90.3° and 102.8° for the tight and wide states, respectively. The angle between the domains' principal axes in the tight and wide states defined the unaltered position of the equatorial (0.84°) and shifted the location of the intermediate (16.05°) and apical (18.72°) domains. Interestingly, the intermediate domain increasingly shifts in respect to the equatorial domain (wide differs from tight by ~15°) and, to a lesser extent, to apical (~5°).

*
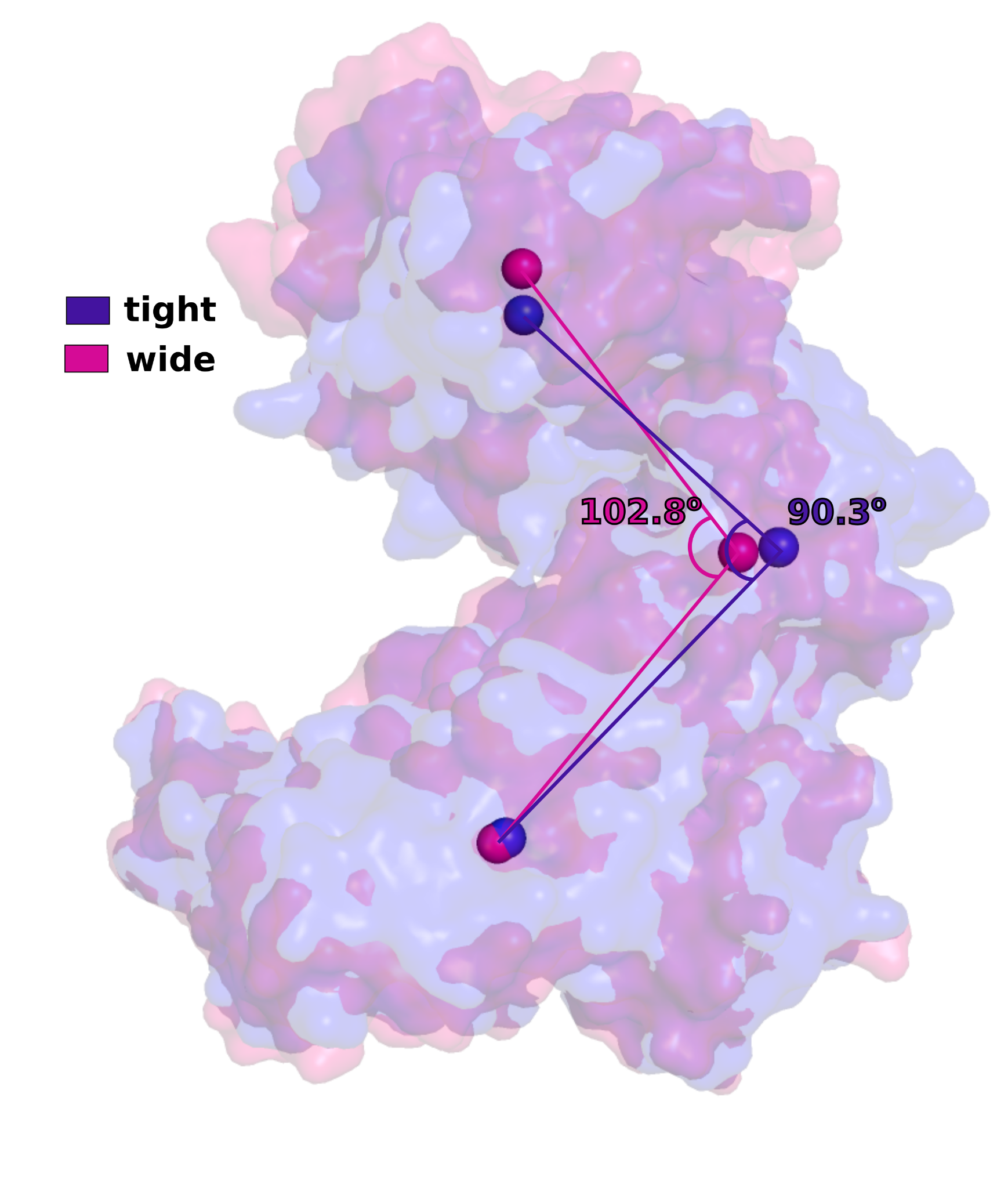

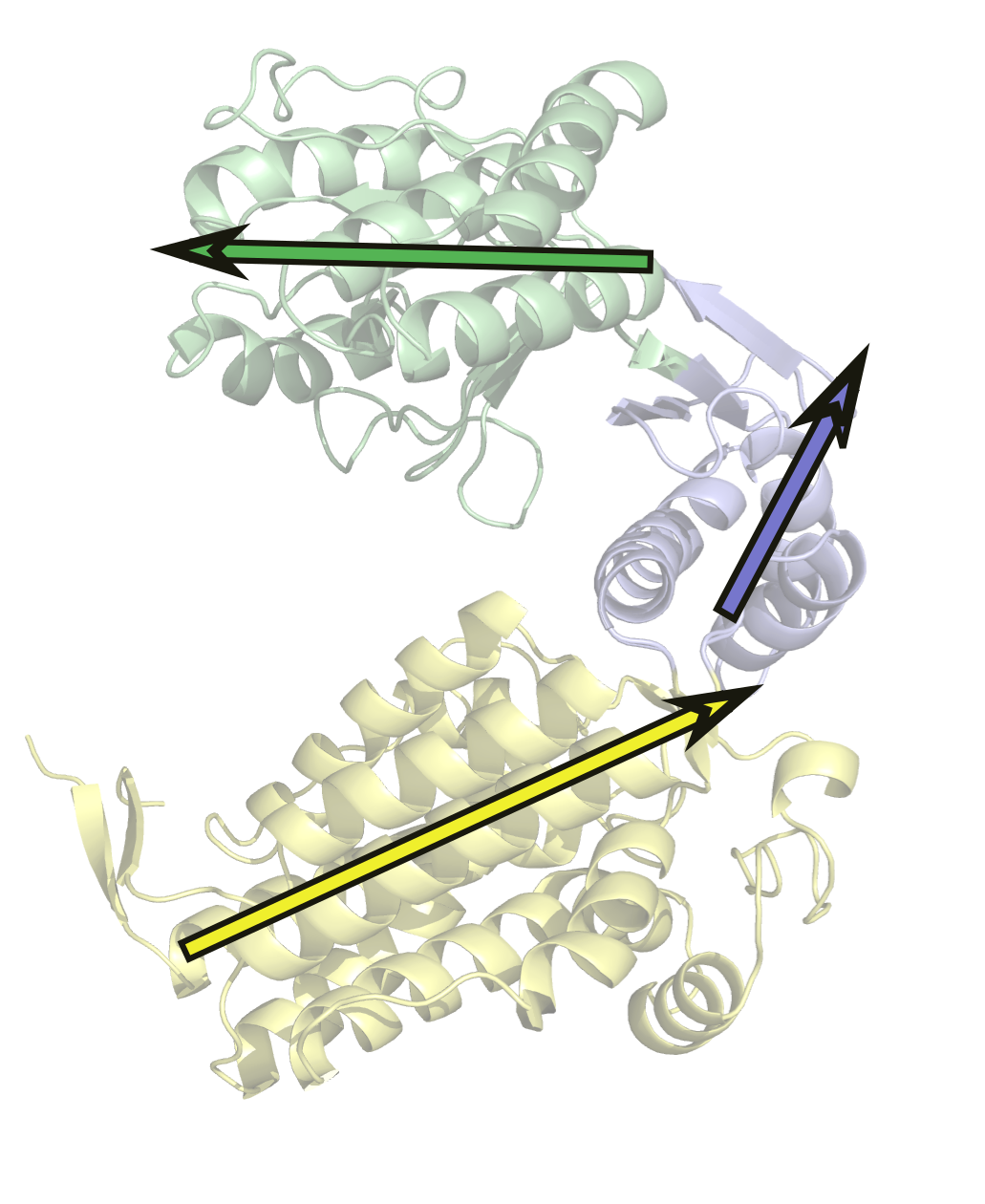
*

*Supplementary Figure S6. Comparison of the structure of the trans-rings in tight and wide conformations.*

*Supplementary Table S1. Comparison of domains’ relative orientation in tight, wide, and R-ADP (4ki8) conformations. Equatorial/Intermediate/Apical - angles between domains’ principal axis (S6 right). COM angle - angle between the centres of domain mass. Because of the conformational diversity in the 4ki8 structure, each subunit is considered.*

|  | | **Equatorial/Intermediate** | **Apical/Intermediate** | **Equatorial/Apical** | **COM angle** |
| --- | --- | --- | --- | --- | --- |
| **Wide** | | **61.8** | **32.8** | **29.3** | **102.8** |
| **Tight** | | **77.3** | **38.3** | **45.4** | **90.3** |
| **4ki8**  **(R-ADP)** | **A** | **63.2** | **40.6** | **36.6** | **103.6** |
|  | **B** | **70.9** | **30.7** | **45.0** | **102.4** |
|  | **C** | **68.1** | **39.7** | **30.8** | **104.1** |
|  | **D** | **51.7** | **37.2** | **14.5** | **114.8** |
|  | **E** | **63.5** | **32.8** | **34.1** | **99.97** |
|  | **F** | **58.7** | **36.8** | **25.1** | **113.8** |
|  | **G** | **55.7** | **21.1** | **35.4** | **113.3** |

*Supplementary Table S2. Comparison of tight and wide conformations with structures deposited to the Protein Data Bank.*

| **PDB ID** | **Publication** | **shape** | **RMSD from wide, A** | **RMSD from tight, A** |
| --- | --- | --- | --- | --- |
| 1aon_subK | The crystal structure of the asymmetric GroEL-GroES-(ADP)7 chaperonin complex | bullet | 5.407 | 2.176 |
| 1pcq_subK | Role of the gamma-phosphate of ATP in triggering protein folding by GroEL-GroES: function, structure and energetics | bullet | 5.313 | 2.114 |
| 1pf9_subK | Role of the gamma-phosphate of ATP in triggering protein folding by GroEL-GroES: function, structure and energetics | bullet | 5.342 | 2.151 |
| 4aaq_subK | ATP-Triggered Conformational Changes Delineate Substrate-Binding and -Folding Mechanics of the Groel Chaperonin | GroEL14 | 5.503 | 1.971 |
| 4aar_subK | ATP-Triggered Conformational Changes Delineate Substrate-Binding and -Folding Mechanics of the Groel Chaperonin | GroEL14 | 5.500 | 1.971 |
| 4aas_subK | ATP-Triggered Conformational Changes Delineate Substrate-Binding and -Folding Mechanics of the Groel Chaperonin | GroEL14 | 5.505 | 1.969 |
| 1svt_subK | Exploring the structural dynamics of the *E. coli* chaperonin GroEL using translation-libration-screw crystallographic refinement of intermediate states | bullet | 5.339 | 2.129 |
| 1sx4_subK | Exploring the structural dynamics of the *E. coli* chaperonin GroEL using translation-libration-screw crystallographic refinement of intermediate states | bullet | 5.379 | 2.172 |
| 1ss8_subA | Exploring the structural dynamics of the *E. coli* chaperonin GroEL using translation-libration-screw crystallographic refinement of intermediate states | GroEL14 | 5.570 | 2.110 |
| 1sx3_subA | Exploring the structural dynamics of the *E. coli* chaperonin GroEL using translation-libration-screw crystallographic refinement of intermediate states | GroEL14 | 4.801 | 1.875 |
| 3zpz_subK | Visualizing Groel/Es in the Act of Encapsulating a Folding Protein | bullet | 5.201 | 1.971 |
| 3zq0_subK | Visualizing Groel/Es in the Act of Encapsulating a Folding Protein | bullet | 5.387 | 2.052 |
| 3zq1_subK | Visualizing Groel/Es in the Act of Encapsulating a Folding Protein | bullet | 4.535 | 2.041 |
| 1gr5_subA | ATP-Bound States of Groel Captured by Cryo-Electron Microscopy | GroEL14 | 6.163 | 2.698 |
| 1gru_subK | ATP-Bound States of Groel Captured by Cryo-Electron Microscopy | bullet | 4.958 | 3.429 |
| 2c7e_subK | ATP-Bound States of Groel Captured by Cryo-Electron Microscopy | GroEL14 | 2.749 | 2.516 |
| 4hel_subA | - |  | 6.480 | 2.940 |
| 2c7c_subK | Allosteric Signalling of ATP Hydrolysis in Groel-Groes Complexes | bullet | 2.996 | 2.961 |
| 4aaq_subA | ATP-Triggered Conformational Changes Delineate Substrate-Binding and -Folding Mechanics of the Groel Chaperonin | GroEL14 | 2.902 | 5.140 |
| 4aar_subA | ATP-Triggered Conformational Changes Delineate Substrate-Binding and -Folding Mechanics of the Groel Chaperonin | GroEL14 | 2.895 | 5.575 |
| 4aau_subA | ATP-Triggered Conformational Changes Delineate Substrate-Binding and -Folding Mechanics of the Groel Chaperonin | GroEL14 | 4.305 | 7.084 |
| 4aau_subK | ATP-Triggered Conformational Changes Delineate Substrate-Binding and -Folding Mechanics of the Groel Chaperonin | GroEL14 | 2.737 | 5.086 |
| 4ab2_subA | ATP-Triggered Conformational Changes Delineate Substrate-Binding and -Folding Mechanics of the Groel Chaperonin | GroEL14 | 4.229 | 6.764 |
| 4ab2_subK | ATP-Triggered Conformational Changes Delineate Substrate-Binding and -Folding Mechanics of the Groel Chaperonin | GroEL14 | 3.720 | 6.366 |
| 4ab3_subA | ATP-Triggered Conformational Changes Delineate Substrate-Binding and -Folding Mechanics of the Groel Chaperonin | GroEL14 | 8.537 | 11.328 |
| 4ab3_subK | ATP-Triggered Conformational Changes Delineate Substrate-Binding and -Folding Mechanics of the Groel Chaperonin | GroEL14 | 3.716 | 6.450 |
| 4aas_subA | ATP-Triggered Conformational Changes Delineate Substrate-Binding and -Folding Mechanics of the Groel Chaperonin | GroEL14 | 7.230 | 10.026 |
| 2c7d_subK | Allosteric Signalling of ATP Hydrolysis in Groel-Groes Complexes | bullet | 2.675 | 3.750 |
| 2c7e_subA | ATP-Bound States of Groel Captured by Cryo-Electron Microscopy | GroEL14 | 4.780 | 6.852 |

**GroEL structure obtained with Cryo-EM**

The same sample that allowed us to reconstruct the structure of the GroEL-ADP14-GroES1 complex contained GroEL free of the GroES. We addressed the question on the nucleotide content of these particles and found that both cis and trans rings contain the additional density in the nucleotide binding pockets. The map resolution was not enough for reliable ligand fitting and direct distinguishing between the ADP and ATP. We fitted the ATP and ADP-bound models (1kp8 and 4ki8 respectively) into the GroEL experimental density with C7 symmetry imposed (Figure S7). The cis ring density clearly fits with the R-state, while the R-ADP fitting reveals significant mismatches between model and density in the apical domain region. The trans ring shows better fit with the R-ADP model, however the trans ring map region has a lower resolution and demonstrates some mismatches for both R and R-ADP models.

*
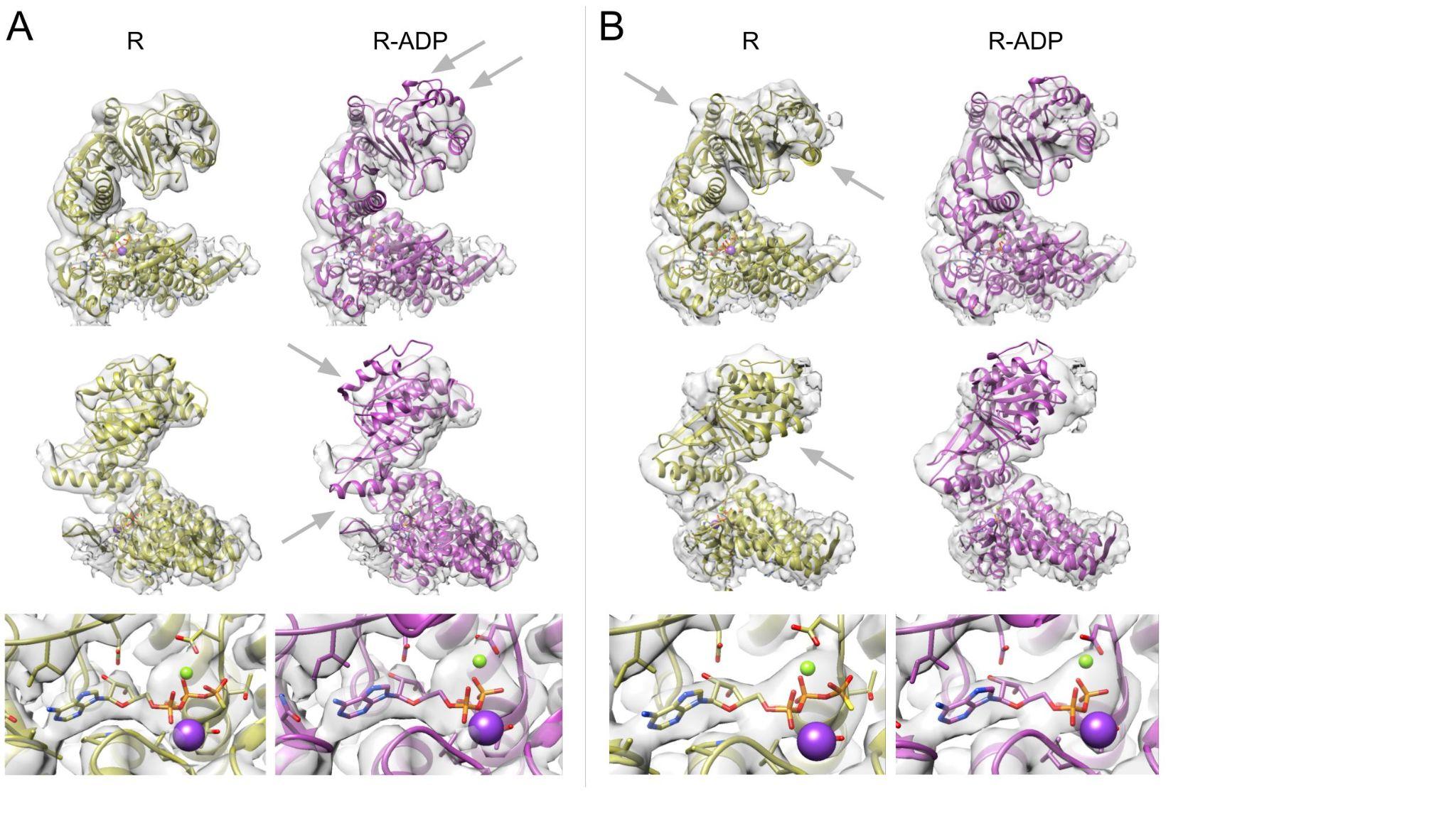
*

*Supplementary Figure S7. Fitting of GroEL R and R-ADP models (1kp8 and 4ki8) into the GroEL experimental density reconstructed with C7 symmetry: A - cis-ring, B - trans-ring. Arrows indicate mismatches between the model and density.*

**ATP/ADP concentration in the samples**

To check the ADP concentration throughout the GroEL-GroES cycle in this study, NADH assay was performed (ATP regenerating assay system)^1,2^**.** The reaction medium contained 50 mM HEPES-NaOH, pH 8.0, 100 mM KCl, 1 mM MgCl_2_, 2.5 mM phosphoenolpyruvate, 125 μM NADH, pyruvate kinase and lactate dehydrogenase (40 units/ml each). The reaction was started by the addition of aliquot containing ADP. Aliquots were taken from the experimental mixture where 1 μM GroEL was co-incubated with 3 μM GroES in Tris-HCl buffer (pH 7.5) containing 10 mM MgCl_2_ and 3 mM ATP at 20° C for 20 min and then concentrated 10 times with a 100 kDa concentrator. Incubation was continued for another 40 min (for 60 min incubation in total). In these conditions concentration of ADP was controlled every 10 minutes (Figure S8). Measurements were performed at 20 °C on Shimadzu UV-1601 UV/VIS Visible spectrophotometer.

*
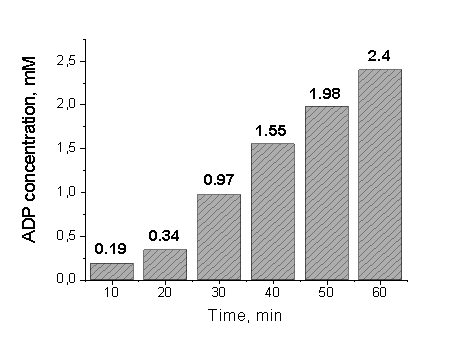
*

*Supplementary Figure S8. Concentration of the ADP in samples incubated for Cryo-EM study*

**References**

1. Scharschmidt, B. F. et al. Validation of a recording spectrophotometric method for measurement of membrane-associated Mg- and NaK-ATPase activity. *J Lab Clin Med.* **93**, 790-799. https://doi.org/10.5555/uri:pii:002221437990091X (1979).

2. Lapashina, A. et al. Residue 249 in subunit beta regulates ADP inhibition and its phosphate modulation in Escherichia coli ATP synthase. *Biochim Biophys Acta Bioenerg.* **1860**, 181–188. https://doi.org/10.1016/j.bbabio.2018.12.003 (2019).
